# KAT/3BP: A Metabolism-Targeting Agent with Single and Combination Activity in Aggressive B-Cell Lymphomas

**DOI:** 10.3390/cancers17122034

**Published:** 2025-06-18

**Authors:** Chiara Tarantelli, Filippo Spriano, Elisa Civanelli, Luca Aresu, Giorgia Risi, Eleonora Cannas, Omar Kayali, Luciano Cascione, Alberto J. Arribas, Anastasios Stathis, Young H. Ko, Francesco Bertoni

**Affiliations:** 1 Institute of Oncology Research, Faculty of Biomedical Sciences, Università Della Svizzera Italiana, 6500 Bellinzona, Switzerland; chiara.tarantelli@ior.usi.ch (C.T.); filippo.spriano@ior.usi.ch (F.S.); elisa.civanelli@ior.usi.ch (E.C.); giorgia.risi17@gmail.com (G.R.); alberto.arribas@ior.usi.ch (A.J.A.); 2 Department of Veterinary Sciences, University of Turin, 10095 Turin, Italy; luca.aresu@unito.it; 3 SIB Swiss Institute of Bioinformatics, 1015 Lausanne, Switzerland; 4 Oncology Institute of Southern Switzerland, Ente Ospedaliero Cantonale, 6500 Bellinzona, Switzerland; 5 Faculty of Biomedical Sciences, Università Della Svizzera Italiana, 6900 Lugano, Switzerland; 6 KoDiscovery, LLC, IMET/Columbus Center, Baltimore, MD 21202, USA

**Keywords:** lymphoma, R-CHOP, 3-Bromopyruvate, HK2, MCT1, cancer, metabolism

## Abstract

Background/Objectives: Reprogramming of the cellular metabolism is a hallmark of cancer, offering therapeutic opportunities to target cancer cell vulnerabilities for therapeutic purposes. 3-Bromopyruvate (3BP) is a small alkylating agent that functions as an anti-metabolite, targeting key substrates in cancer metabolism and demonstrating antitumor activity across multiple cancer types. However, unformulated 3BP is associated with significant toxicity. This study investigates the efficacy of KAT/3BP, a clinical derivative of 3BP currently in phase 1 trials for hepatocellular carcinoma, in preclinical lymphoma models. Results: *In vitro*, KAT/3BP exhibited cytotoxic activity across 12 lymphoma cell lines—including diffuse large B-cell lymphoma and mantle cell lymphoma—with a median IC_50_ of 3.7 μM. It also remained effective against lymphoma cell lines with acquired resistance to FDA-approved therapies. *In vivo*, treatment with KAT/3BP led to reduced tumor size in a syngeneic mouse model, with the combination of oral and intratumoral administration showing the greatest efficacy. Furthermore, KAT/3BP demonstrated synergistic activity when combined with standard lymphoma therapies such as bendamustine and R-CHOP. Conclusions: Our findings highlight the potential of KAT/3BP as a novel therapeutic option, either as a single agent or in combination regimens, for treating lymphomas.

## Simple Summary

Cancer cells often rely on an altered metabolism to grow and survive. Scientists are looking for ways to target this peculiarity and stop cancer from spreading. In this study, we tested a new drug called KAT/3BP, designed to block cancer cell metabolism. We found that it killed cancer cells derived from different types of lymphoma, including some that had become resistant to existing treatments. The drug also worked in mice with transplanted lymphoma cells, especially when given by mouth and directly into the tumor. In some cases, tumors completely disappeared without causing severe side effects. We also tested KAT/3BP with standard cancer treatments and found that the combination was more effective than either treatment alone. Our work supports future testing of this drug in individuals with lymphoma to improve the currently available treatment options.

## 1. Introduction

Reprogramming of the cellular metabolism is a hallmark of cancer and presents therapeutic opportunities to exploit cancer-specific vulnerabilities [1,2]. 3-Bromopyruvate (3BP) is a small, highly reactive alkylating agent derived from the bromination of pyruvate [3,4,5,6,7]. Due to its high structural similarity with pyruvate (Krebs cycle), lactate (Warburg effect), and acetate (lipogenesis), 3BP functions as an anti-metabolite, interfering with key metabolic substrates essential for cancer cell survival. In addition, as a potent alkylating agent, 3BP can modify multiple proteins, including glycolytic and mitochondrial enzymes. Under acidic extracellular pH, 3BP is transported into cancer cells via monocarboxylate transporter 1 (MCT1), inhibiting glycolysis by covalently modifying hexokinase II (HK2). This leads to HK2 dissociation from mitochondria, release of apoptosis-inducing factor (AIF), and induction of apoptosis [7]. Preclinical studies have demonstrated the antitumor activity of 3BP, both as a monotherapy and in combination regimens, in various solid tumors, multiple myeloma, and leukemias [8,9,10,11,12,13,14]. In lymphoma, antitumor effects have been reported *in vitro* and *in vivo* using the Burkitt lymphoma Raji cell line [15,16] and a syngeneic mouse model of T-cell lymphoma [17,18]. In the latter, tumor regression was accompanied by increased circulating CD4^+^, CD8^+^, and NK cells, enhanced tumor-associated macrophage infiltration, and reduced local immunosuppression [17]. Despite the high metabolic activity of cancer cells, which should allow a preferential effect on neoplastic than healthy cells, unformulated 3BP has been associated with severe toxicities, including fatal outcomes [19,20]. To address this, novel formulations —liposomes, polyethylene glycol (PEG), PEGylated liposomes (stealth liposomes), perillyl alcohol formulations, and others—have been developed to enhance delivery and reduce toxicity [9,19,21]. Notably, a patient with fibrolamellar hepatocellular carcinoma has been safely treated with formulated 3BP via transcatheter arterial chemoembolization [21]. Two clinical 3BP derivatives, KAT-101 and KAT-201, have been developed for oral and intratumoral (IT) administration, respectively (National Cancer Institute Thesaurus Codes C193479 and C193479), and are currently under early clinical evaluation in patients with hepatocellular carcinoma (NCT05603572).

Importantly, HK2—a key 3BP target—is a known metabolic driver in diffuse large B-cell lymphoma (DLBCL) cell lines and patients [22]. Furthermore, immunohistochemical analysis of 120 DLBCL specimens revealed universal expression of MCT1, the 3BP transporter [23]. Given the limited preclinical data on 3BP-based therapies in lymphoma, we investigated the antitumor activity of the 3BP derivative KAT/3BP in lymphoma models, including cell lines with secondary resistance to FDA-approved agents and a syngeneic mouse model.

## 2. Materials and Methods

### 2.1. Cell Lines

B-cell lymphoma-derived cell lines (TMD8, RCK8, U2932, SUDHL2; OCILY19, WSU-DLCL2, DOHH2, TOLEDO; MINO, REC1, GRANTA519, and Z138) were cultured in the appropriate medium supplemented with fetal bovine serum (10% or 20%) and penicillin–streptomycin–neomycin (≈5000 units penicillin, 5 mg streptomycin, and 10 mg neomycin/mL; Sigma-Aldrich, Darmstadt, Germany). Cell line identities were confirmed by short tandem repeat DNA fingerprinting using the Promega GenePrint 10 System kit (B9510). Cells were periodically tested for Mycoplasma negativity using the MycoAlert Mycoplasma Detection Kit (Lonza, Visp, Switzerland).

### 2.2. Compounds

KAT/3BP was provided by KoDiscovery, LLC (Baltimore, MD, USA). Tazemetostat, ibrutinib, lenalidomide, bendamustine, copanlisib, venetoclax, vorinostat, doxorubicin, vincristine, and prednisolone were purchased from Selleckchem (Houston, TX, USA). Rituximab was purchased from Roche (Basel, Switzerland), and 4-hydroperoxy-cyclophosphamide from Santa Cruz Biotechnology (Heidelberg, Germany).

### 2.3. In Vitro Cytotoxic Activity

Cells were manually seeded in 96-well plates at a concentration of 50,000 cells/mL (10,000 cells in each well). Treatments were performed manually. After 72 h, cell viability was determined using 3-(4,5-dimethyl-thiazol-2-yl)-2,5-diphenyltetrazolium bromide (MTT), and the reaction was stopped after 4 h with sodium dodecyl sulfate lysis buffer.

For combination studies, cells were exposed (72 h) to seven increasing concentrations of the two agents, either alone or combined, followed by an MTT assay. ZIP, HAS, Loewe, and Bliss parameters were calculated for a fixed dose of KAT/3BP, giving antiproliferative activity between 70 and 10% using SynergyFinder software version 3.0 [24,25].

For R-CHOP treatment, rituximab was used at a fixed dose (20 μg/mL, representing clinically recommended serum levels [26]), and CHOP was prepared as a mix reflecting the clinical ratios of the drugs (83.2%, 4-hydroperoxy-cyclophosphamide; 5.54%, doxorubicin; 0.16%, vincristine; 11.1%, prednisolone) [27,28].

### 2.4. Cell Cycle and Apoptosis Assessment

The apoptosis induction and cell cycle analysis was performed after 24, 48, and 72 h of drug exposure at 5 μM or DMSO. For apoptosis analysis, cells were stained with Annexin V-FITC, and, after 10 min of incubation, cells were washed and incubated with propidium iodide (PI, ThermoFisher Scientific, Waltham, MA, USA). The percentage of apoptotic cells (Annexin V positive/PI negative and Annexin V positive/PI positive) was assessed. For cell cycle analysis, cells were fixed and permeabilized with 70% cold ethanol and stained with PI and RNase (Sigma Aldrich, Buchs, Switzerland) after 24 h of incubation. The percentage of cells in sub-G0, G1, S, or G2-M phases was assessed. Data were acquired at the FACSCanto I instrument (BD Biosciences, Allschwil, Switzerland) and analyzed using FlowJo software version 11 (TreeStar Inc., Ashland, OR, USA).

### 2.5. In Vivo Syngeneic Mouse Models

KAT/3BP was prepared by dissolving 3BP in a buffer system based on sodium phosphate and sodium citrate for oral, IP, and IT deliveries. Mice maintenance and animal experiments were performed under the institutional guidelines established by the Cantonal Committee for Animal Experimentation (CCEA) of Università della Svizzera Italiana (USI) (protocol code 30551 and date of approval 6 October 2023). BALB/cAnNCrl mice were obtained from Charles River (Calco, Lecco, Italy). Tumors were established by injecting A20 murine lymphoma cells (5 × 10^6^ cells/mouse, 100 µL of PBS) into the left flanks of female BALB/c mice (6–8 weeks of age, approximately 20 g of body weight). Treatments started once tumor volume reached approximately 60 mm^3^ for pilot treatments and 140 mm^3^ for combination studies, as an average for each group. A stratified random allocation was designed, and the blocks were based on average tumor volume and mouse weight, with each animal representing an experimental unit. The G-power software (version 3.1) was used to determine the number of animals per group. Tumor volume was measured three times per week using a digital caliper, and animal body weight was measured three times per week throughout this study. The animal status was carefully evaluated during housing and treatments by measuring the Cumulative Condition Scores. Mice were sacrificed once tumor volumes reached 2000 mm^3^ and/or when several parameters were scored with a high severity degree (body weight loss, body condition score, physical condition, behavior, hydration, respiration). For intratumoral (IT) administration, the endpoint was set at 1500 mm^3^. Tumor samples were fixed in buffered formalin and examined histologically using hematoxylin and eosin staining. A semi-quantitative scoring system, ranging from 0 to 3, was employed to assess the extent of necrosis, corresponding to the percentage of tissue area affected by the necrotic process. The scoring criteria were as follows: score 0 indicated no necrosis present; score 1 represented 1–10% of the tissue involved; score 2 denoted 11–20% of the tissue involved; and score 3 signified approximately 25% of the tissue affected. The assessment was conducted across ten microscopic fields at 40× magnification.

### 2.6. Statistical Analysis

Statistical analyses, including IC50s determination, were conducted using Prism software v10.2.3 (GraphPad Software, La Jolla, CA, USA). Statistical significance was determined by a two-tailed unpaired Student’s *t*-test or as described in the figure legends. A *p* value < 0.05 was considered statistically significant. For *in vivo* experiments, simple linear regression analysis was applied to extrapolate the best-fit slopes from each treatment using Prism software v10.2.3.

## 3. Results

### 3.1. KAT/3BP as a Single Agent Is Cytotoxic in Lymphoma Cell Lines

**Figure 1:**
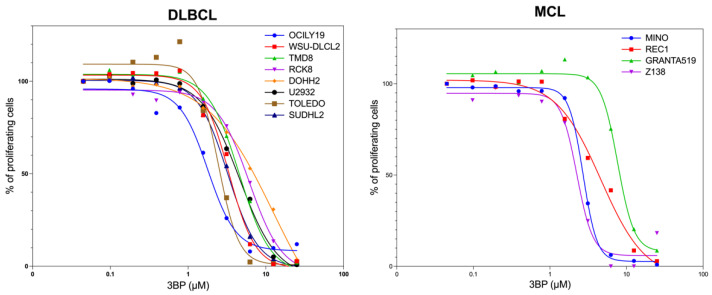
Antiproliferative effect of KAT/3BP in DLBCL and MCL subtypes of lymphoma. The dose–response curves in eight diffuse large B cell lymphoma (**left** panel) and four mantle cell lymphoma cell lines (**right** panel) treated with increasing KAT/3BP compound concentrations. An MTT assay was performed to evaluate the antitumoral activity of the drug. Averages of at least two replicates are shown in the figure.

Twelve lymphoma cell lines derived from activated B-cell-like diffuse large B-cell lymphoma (ABC DLBCL) (n = 4; TMD8, RCK8, U2932, SUDHL2), germinal center B-cell like (GCB) DLBCL (n = 4; OCILY19, WSU-DLCL2, DOHH2, TOLEDO), and mantle cell lymphoma (MCL) (n = 4; MINO, REC1, GRANTA519, Z138) were exposed to increasing concentrations of the 3BP clinical derivative KAT/3BP for 72 h (Figure 1). The median IC50 across all the cell lines was 3.7 μM, with no differences based on the histotypes.

Cell cycle analyses in one DLBCL (Toledo) and one MCL (Z138) cell line exposed to KAT/3BP at 5 μM or DMSO as a control for 24, 48, and 72 h showed a time-dependent increase in the percentage of cells in sub-G0 (Supplementary Figure S1A). Thus, an Annexin V test showed an apoptosis induction in both cell lines already at 24 h of exposure to KAT/3BP at 5 μM (Supplementary Figure S1B).

### 3.2. KAT/3BP as a Single Agent Exerts Anti-Lymphoma Activity in Models of Secondary Resistance to FDA-Approved Agents

**Figure 2:**
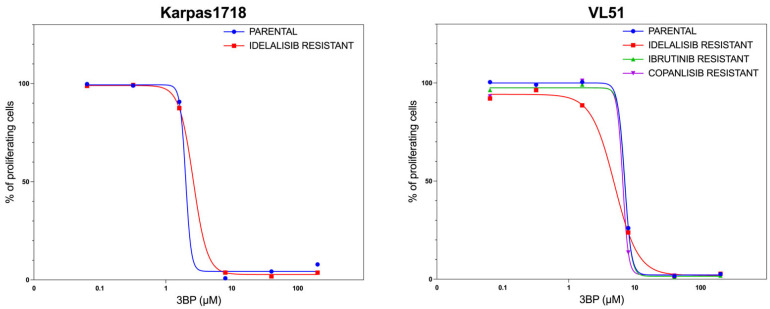
Antiproliferative effect of KAT/3BP in MZL models of secondary resistance to FDA-approved agents. Marginal zone lymphoma cell lines (parental and resistant to idelalisib, ibrutinib, and copanlisib) were treated with increasing KAT/3BP compound concentrations. An MTT assay was performed to evaluate the antitumoral activity of the drug. Averages of at least two replicates are shown in the figure.

Based on the activity observed in DLBCL and MCL cell lines, we also tested the compound in two marginal zone lymphoma (MZL) cell lines (Karpas1718 and VL51) and their derivatives with acquired resistance to PI3K inhibitors, BTK inhibitors, and BCL2 inhibitors [29,30,31,32]. KAT/3BP showed a dose-dependent antiproliferative activity in the two MZL cell lines and the resistant cells (Figure 2).

### 3.3. KAT/3BP Has In Vivo Antitumor Activity in a Syngeneic Mouse Model

We then validated the *in vitro* results using a murine syngeneic model (A20 lymphoma cells, BALB/c mice). First, we confirmed that KAT/3BP was also *in vitro* active in the A20 lymphoma cells (Supplementary Figure S2).

The mice were treated with KAT/3BP as oral, intraperitoneal (IP), or IT delivery routes or with a buffer as a control for 28 days (Supplementary Figure S3A). All the mice in the control groups survived up to day 26 for the IP administration group and day 19 for the PO and IT groups.

**Figure 3:**
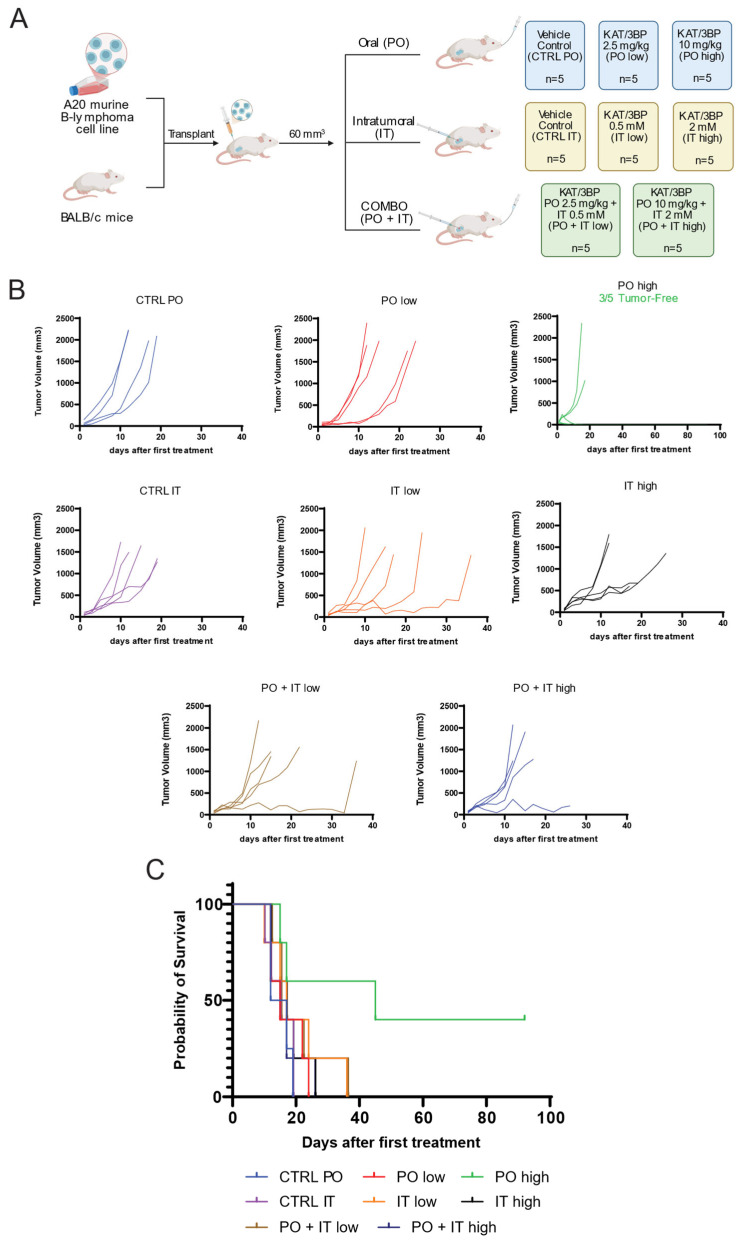
Assessment of KAT/3BP anti-lymphoma activity in an *in vivo* syngeneic model. BALB/c mice were subcutaneously injected with the murine lymphoma cell line A20. Mice were treated with vehicle (SFB) by oral or intratumoral (IT) injection, with 2.5 mg/kg and 10 mg/kg per os (low and high PO, respectively), with 0.5 mM and 2 mM by IT (IT low and high, respectively), with the combination of 2.5 mg/kg PO plus 0.5 mM IT, or with the combination of 10 mg/kg PO and 2 mM IT (PO + IT low and PO + IT high, respectively). All groups were composed of five mice, and the end of the experiment was set at day 92 for mice with complete tumor regression. (**A**) Schematic representation of the experimental plan. (**B**) Graphs showing tumor volume in mm^3^ for each animal in each group. (**C**) Survival for each group.

Treatments with oral or IT KAT/3BP administration determined reduced tumor size compared to the control groups (Figure 3B,C, Supplementary Figure S3). This effect was further highlighted by the slope values extrapolated with a linear regression model (Table 1). Treatments with oral administration at 10 mg/kg dose led to a total tumor reduction in three mice out of five; two were still alive at day 92, and one was sacrificed on day 45. The latter had a relapse in the upper part of the thorax. One mouse in the IT-low group and another one in the PO + IT-low group were sacrificed due to tumor growth, which occurred much later than what was seen in the control group.

Necrosis was observed in both the periphery and the tumor’s center, exhibiting a multifocal distribution. The PO + IT high, PO high, and IT high groups (one sample each) were characterized by necrosis. However, a score of 3, indicating the highest level of necrosis, was only observed in both the IT high and PO + IT high groups (Supplementary Figure S4).

IP administration was not tolerated at a high dose of 10 mg/kg, with 10–15% body weight loss after three days of treatment, dehydration, and a cumulative condition score at the maximum limit (Supplementary Figure S5A–C). The IP low-dose group showed a 5% body weight loss after three days and recovered after eating soft food in the cage. Treatment was not effective in tumor reduction.

**Figure 4:**
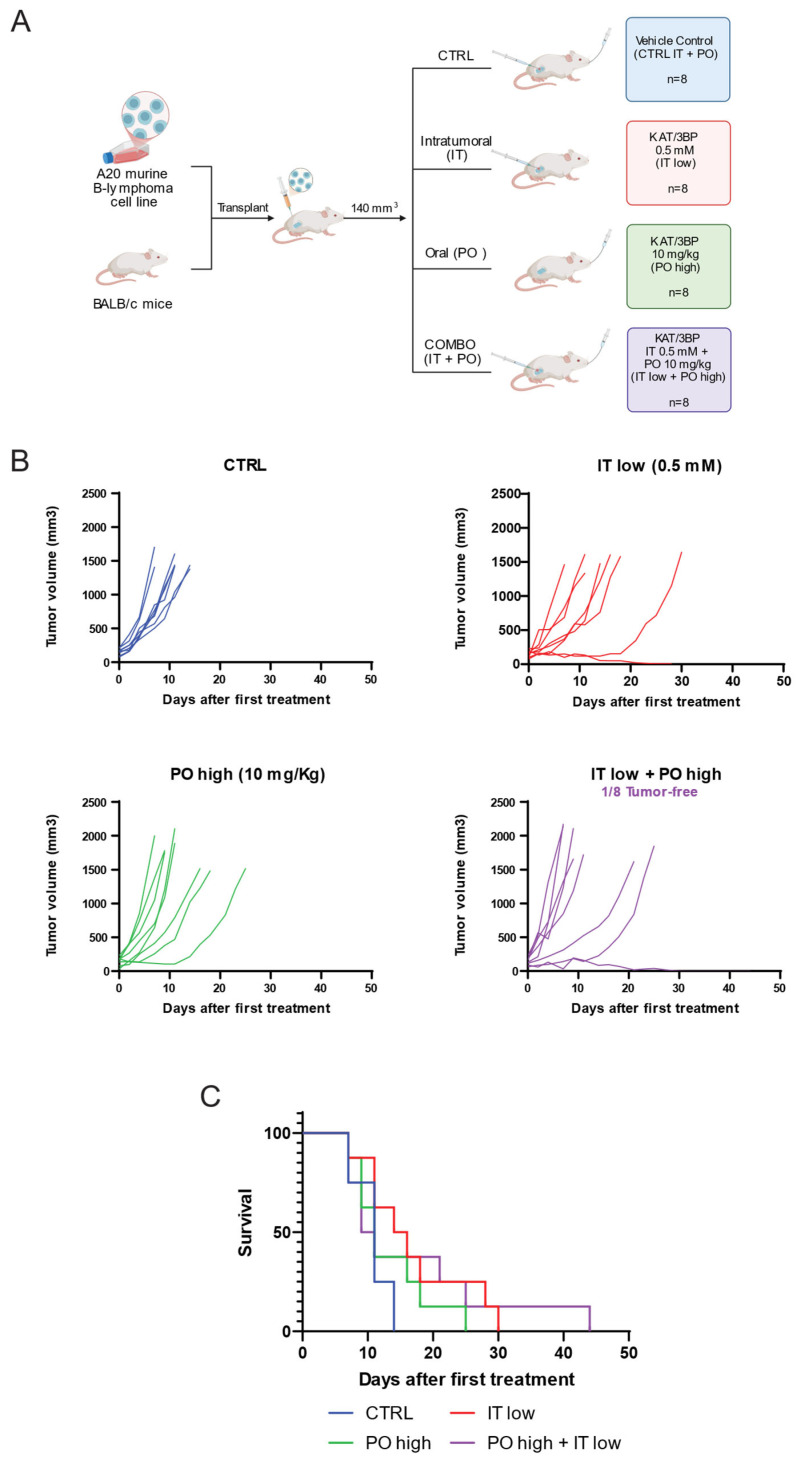
Assessment of KAT/3BP anti-lymphoma activity in PO and IT administration as single and in combination. BALB/c mice were subcutaneously injected with the murine lymphoma cell line A20. Mice were treated with vehicle (SFB) by oral or intratumoral (IT) injection, with 10 mg/kg per os (PO high), with 0.5 mM by IT (IT low), with the combination of PO high plus IT low, or with the combination of the two (PO high + IT low). All groups were composed of 8 mice, and the end of the experiment was set at day 92 for mice with complete tumor regression. (**A**) Schematic representation of the experimental plan. (**B**) Graphs showing tumor volume in mm^3^ for each animal in each group. (**C**) Survival for each group. End of the experiment: D44.

We then evaluated the combination of oral and IT KAT/3BP. Based on the pilot experiment results, an a priori power analysis for a two-group two-tailed *t*-test determined the number of animals. Effect size of 1.56, type I error of 0.05, power of 0.8, and allocation ratio of 1:1 were considered. Groups of eight mice each were treated orally and intratumorally for 28 days with the vehicle, oral KAT/3BP administered at a high dosage (10 mg/kg), IT KAT/3BP at a low dosage (0.5 mM), and the combination of the two (Figure 4A). Treatments with oral plus IT KAT/3BP decreased tumor volume growth compared to the other groups, with complete tumor reduction in one mouse out of eight combination groups (Figure 4B, Supplementary Figure S6A). Based on the slope calculated with linear regression mode, a tumor reduction higher than the control vehicle was observed in all the treatment groups, especially in the combination group (Table 2).

The survival graph showed that all the treated groups of mice, regardless of the administration route, had better survival than the vehicle group (Figure 4C). In the combination group, one mouse survived up to day 44. The experiment was stopped due to license limitations, which prevented single-housed animals from being kept. The animal was visually inspected for any presence of a tumor, and the absence of any cancer was confirmed. One mouse in the IT group developed a second tumor and was sacrificed on day 28 when the maximum volume was unacceptable.

Tumor ulcerations were detected in two mice in the vehicle group (PO high + IT low administration), four in the IT group, one in the PO group, and two mice in the combination. The mice’s weight was monitored, and the drug did not induce weight loss of more than 5% (Supplementary Figure S6B).

### 3.4. KAT/3BP-Based Combinations Are Active in Lymphoma Cell Lines

**Figure 5:**
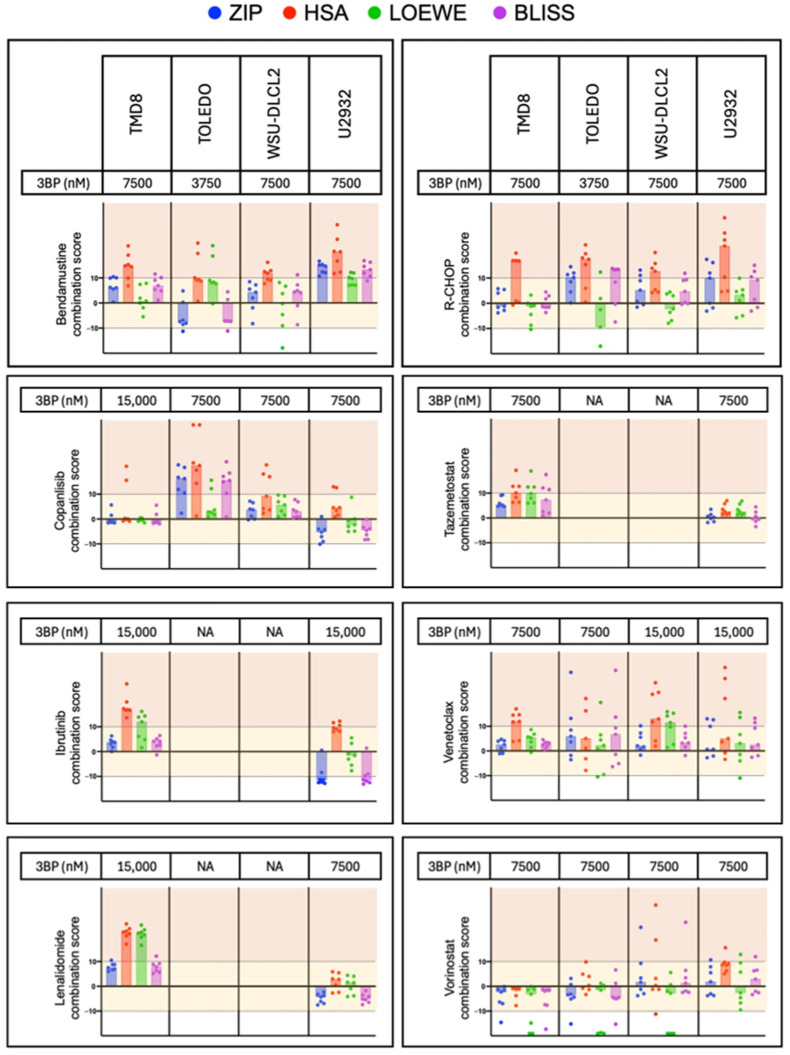
KAT/3BP-containing combinations. The figure shows ZIP, HSA, Loewe, and Bliss combination scores obtained by combining KAT/3BP at a fixed dose with a second compound at increasing doses. Values below −10 indicate no benefit from combining the two drugs; values between −10 and 10 indicate additivity (yellow area in the graph), while values above 10 indicate synergism (red area in the graph).

Based on the demonstration of *in vitro* and *in vivo* single-agent activity, we explored possible KAT/3BP-based combinations. We tested FDA-approved agents or molecules targeting critical pathways in lymphoma. Cell lines were exposed for 72 h to a fixed dose of KAT/3BP, increasing concentrations of the second drug as single agents and combined. The latter included the EZH2 inhibitor tazemetostat, BTK inhibitor ibrutinib, the cereblon E3 ligase modulator lenalidomide, the DNA binding agent bendamustine, the PI3K α/δ inhibitor copanlisib, the BCL2 inhibitor venetoclax, the HDAC inhibitor vorinostat, and the immuno-chemotherapeutic R-CHOP. Combinations were tested in cell lines derived from GCB (TOLEDO, WSU-DLCL2) and ABC DLBCL (TMD8, U2932). Ibrutinib and lenalidomide were tested only in cell lines derived from ABC DLBCL, while tazemetostat in GCB DLBCL is the subtype in which the drugs have reported clinical activity. Figure 5 represents the combination results. For each combination, we calculated four different synergism parameters: HSA, Bliss, Lowe, and ZIP. Overall, KAT/3BP was beneficial with all the tested compounds. In more detail, we observed synergism in at least one of the four parameters, in combination with R-CHOP in all four DLBCL cell lines. Bendamustine plus KAT/3BP were synergistic in all except TOLEDO. Tazemetostat plus KAT/3BP were synergistic in the TOLEDO. Combinations with lenalidomide, ibrutinib, and venetoclax were synergistic in TMD8.

## 4. Discussion

We demonstrated that KAT/3BP exhibits both *in vitro* and *in vivo* cytotoxic activity in lymphoma models as a single agent, with efficacy maintained in models harboring secondary resistance to BTK, BCL2, and PI3K inhibitors. Furthermore, we showed that combining KAT/3BP with other anti-lymphoma agents enhanced therapeutic outcomes. *In vitro*, KAT/3BP displayed potent cytotoxicity in the low micromolar range across cell lines derived from three aggressive B-cell lymphoma subtypes —ABC DLBCL, GCB DLBCL, and MCL—for which many patients still lack effective treatment options [33,34,35]. Notably, the responsive models are known to exhibit limited sensitivity to the CD19-targeting antibody–drug conjugate loncastuximab tesirine and the standard R-CHOP regimen (e.g., U2932 and SU-DHL-2) [36], as well as to the CD79B-targeting agent polatuzumab vedotin (e.g., RCK8, SU-DHL-2) [37].

We also observed KAT/3BP activity in two MZL cell lines and, importantly, in their derivatives with acquired resistance to BTK, PI3K, and BCL2 inhibitors. This is consistent with previous studies reporting apoptosis induction by 3BP in colorectal cancer models resistant to EGFR inhibitors [14]. The ability of KAT/3BP to remain effective in resistant models highlights its potential to overcome common mechanisms of therapeutic resistance, a significant challenge in lymphoma treatment.

These *in vitro* findings were corroborated *in vivo* using a syngeneic lymphoma mouse model. Notably, mice were not fasted before KAT/3BP treatment, despite evidence suggesting that fasting may potentiate 3BP’s antitumor effects [38,39,40]. Oral administration of KAT/3BP at 10 mg/kg significantly reduced tumor burden, with three mice remaining tumor-free through day 45 and two through day 92. The addition of IT KAT/3BP further enhanced tumor regression, suggesting that a combined oral and local delivery strategy may optimize therapeutic efficacy. In contrast, intraperitoneal administration at the same dose was acutely toxic, resulting in significant body weight loss and necessitating early sacrifice of affected animals.

The observed antitumor effects of KAT/3BP were predominantly cytotoxic, as evidenced by apoptosis induction *in vitro* and necrosis in some tumors *in vivo*. Similar necrotic responses have been described in other tumor models treated with 3BP [41,42,43], though the administration route may also have influenced these outcomes.

Lastly, we demonstrated that combining KAT/3BP with established lymphoma therapies, particularly chemotherapy-based regimens, further enhanced antitumor efficacy. Specifically, synergistic effects were observed with bendamustine and R-CHOP. However, the benefit was more selective for targeted therapies, as only specific models and agents showed improvement upon the addition of KAT/3BP.

## 5. Conclusions

In summary, our data show that KAT/3BP exhibits significant *in vitro* and *in vivo* antitumor activity in lymphoma models, including those with primary resistance to R-CHOP and antibody–drug conjugates, as well as secondary resistance to BTK and PI3K inhibitors. Its combination with standard therapies, particularly chemotherapies, further enhances its therapeutic potential. These findings support the extension of ongoing early-phase clinical evaluation of KAT/3BP—currently underway in hepatocellular carcinoma—to include patients with lymphoma.

## Figures and Tables

**Table 1:** Estimated slopes for each group under a single administration route of 3BP in an *in vivo* syngeneic model. The table shows the slopes calculated following a linear regression model.

	CTRL PO	PO Low	PO High	CTRL IT	IT Low	IT High	PO + IT Low	PO + IT High
Best-fit values Slope	101.8	67.84	−2.610	68.24	18.71	39.26	13.95	22.39

**Table 2:** Slope values are extrapolated by a linear regression model in PO and IT KAT/3BP administration as single and combination routes. The table shows the slopes calculated following a linear regression model.

	CTRL PO	PO High	IT Low	PO High + IT Low
Best-fit values Slope	104.2	19.80	54.30	−0.3001

## Data Availability

All data generated or analyzed during this study are included in this published article and its Supplementary Information Files.

## Supplementary Materials

The following supporting information can be downloaded at: https://www.mdpi.com/article/10.3390/cancers17122034/s1, Figure S1: The activity of KAT/3BP on lymphoma cell lines, one diffuse large B cell lymphoma (TOLEDO), and one mantle cell lymphoma (Z138); Figure S2: Antiproliferative effect of 3BP in A20 murine cell lymphoma model; Figure S3: Assessment of KAT/3BP anti-lymphoma activity in *in vivo* syngeneic model; Figure S4: Histological representation of tumor necrosis scores; Figure S5: Activity of KAT/3BP administered by intraperitoneal injection in a syngeneic model; Figure S6: Assessment of KAT/3BP anti-lymphoma activity in PO and IT administration as single and in combination; Table S1: Compounds combined with KAT/3BP with their respective target and range of concentrations used; Table S2: Overall experimental design to assess the efficacy of KAT/3BP via various delivery routes (oral, IT, and IP) in tumor-bearing syngeneic mice; Table S3: Overall experimental design to assess the efficacy of KAT/3BP via various delivery routes in combination (PO, IT, combination) in tumor-bearing syngeneic mice.

## References

[1] Teicher B.A., Linehan W.M., Helman L.J. (2012). Targeting Cancer Metabolism. Clin. Cancer Res..

[2] Hanahan D. (2022). Hallmarks of Cancer: New Dimensions. Cancer Discov..

[3] Pedersen P.L. (2012). 3-bromopyruvate (3BP) a fast acting, promising, powerful, specific, and effective “small molecule” anti-cancer agent taken from labside to bedside: Introduction to a special issue. J. Bioenerg. Biomembr..

[4] Cardaci S., Desideri E., Ciriolo M.R. (2012). Targeting aerobic glycolysis: 3-bromopyruvate as a promising anticancer drug. J. Bioenerg. Biomembr..

[5] Rodrigues-Ferreira C., da Silva A.P.P., Galina A. (2012). Effect of the antitumoral alkylating agent 3-bromopyruvate on mitochondrial respiration: Role of mitochondrially bound hexokinase. J. Bioenerg. Biomembr..

[6] Shoshan M.C. (2012). 3-bromopyruvate: Targets and outcomes. J. Bioenerg. Biomembr..

[7] Azevedo-Silva J., Queirós O., Baltazar F., Ułaszewski S., Goffeau A., Ko Y.H., Pedersen P.L., Preto A., Casal M. (2016). The anticancer agent 3-bromopyruvate: A simple but powerful molecule taken from the lab to the bedside. J. Bioenerg. Biomembr..

[8] Majkowska-Skrobek G., Augustyniak D., Lis P., Bartkowiak A., Gonchar M., Ko Y.H., Pedersen P.L., Goffeau A., Ułaszewski S. (2014). Killing multiple myeloma cells with the small molecule 3-bromopyruvate: Implications for therapy. Anticancer Drugs..

[9] Chapiro J., Sur S., Savic L.J., Ganapathy-Kanniappan S., Reyes J., Duran R., Thiruganasambandam S.C., Moats C.R., Lin M., Luo W. (2014). Systemic delivery of microencapsulated 3-bromopyruvate for the therapy of pancreatic cancer. Clin. Cancer Res..

[10] Gan L., Xiu R., Ren P., Yue M., Su H., Guo G., Xiao D., Yu J., Jiang H., Liu H. (2016). Metabolic targeting of oncogene MYC by selective activation of the proton-coupled monocarboxylate family of transporters. Oncogene.

[11] Pichla M., Sroka J., Pienkowska N., Piwowarczyk K., Madeja Z., Bartosz G., Sadowska-Bartosz I. (2019). Metastatic prostate cancer cells are highly sensitive to 3-bromopyruvic acid. Life Sci..

[12] Skaripa-Koukelli I., Hauton D., Walsby-Tickle J., Thomas E., Owen J., Lakshminarayanan A., Able S., McCullagh J., Carlisle R.C., Vallis K.A. (2021). 3-Bromopyruvate-mediated MCT1-dependent metabolic perturbation sensitizes triple negative breast cancer cells to ionizing radiation. Cancer Metab..

[13] Sołek P., Mytych J., Łannik E., Majchrowicz L., Koszła O., Koziorowska A., Koziorowski M. (2022). Cancer on-target: Selective enhancement of 3-bromopyruvate action by an electromagnetic field in vitro. Free Radic. Biol. Med..

[14] Mu M., Zhang Q., Zhao C., Li X., Chen Z., Sun X., Yu J. (2023). 3-Bromopyruvate overcomes cetuximab resistance in human colorectal cancer cells by inducing autophagy-dependent ferroptosis. Cancer Gene Ther..

[15] Chen Z., Zhang H., Lu W., Huang P. (2009). Role of mitochondria-associated hexokinase II in cancer cell death induced by 3-bromopyruvate. Biochim. Biophys. Acta.

[16] Schaefer N.G., Geschwind J.F., Engles J., Buchanan J.W., Wahl R.L. (2012). Systemic administration of 3-bromopyruvate in treating disseminated aggressive lymphoma. Transl. Res..

[17] Yadav S., Pandey S.K., Goel Y., Kujur P.K., Maurya B.N., Verma A., Kumar A., Singh R.P., Singh S.M. (2018). Protective and recuperative effects of 3-bromopyruvate on immunological, hepatic and renal homeostasis in a murine host bearing ascitic lymphoma: Implication of niche dependent differential roles of macrophages. Biomed. Pharmacother..

[18] Yadav S., Pandey S.K., Kumar A., Kujur P.K., Singh R.P., Singh S.M. (2017). Antitumor and chemosensitizing action of 3-bromopyruvate: Implication of deregulated metabolism. Chem.Biol. Interact..

[19] El Sayed S.M. (2018). Enhancing anticancer effects, decreasing risks and solving practical problems facing 3-bromopyruvate in clinical oncology: 10 years of research experience. Int. J. Nanomed..

[20] Feldwisch-Drentrup H. (2016). Candidate cancer drug suspected after death of three patients at an alternative medicine clinic. Science.

[21] Ko Y.H., Verhoeven H.A., Lee M.J., Corbin D.J., Vogl T.J., Pedersen P.L. (2012). A translational study “case report” on the small molecule “energy blocker” 3-bromopyruvate (3BP) as a potent anticancer agent: From bench side to bedside. J. Bioenerg. Biomembr..

[22] Bhalla K., Jaber S., Nahid M.N., Underwood K., Beheshti A., Landon A., Bhandary B., Bastian P., Evens A.M., Haley J. (2018). Role of hypoxia in Diffuse Large B-cell Lymphoma: Metabolic repression and selective translation of HK2 facilitates development of DLBCL. Sci. Rep..

[23] Noble R.A., Bell N., Blair H., Sikka A., Thomas H., Phillips N., Nakjang S., Miwa S., Crossland R., Rand V. (2017). Inhibition of monocarboxyate transporter 1 by AZD3965 as a novel therapeutic approach for diffuse large B-cell lymphoma and Burkitt lymphoma. Haematologica.

[24] Yadav B., Wennerberg K., Aittokallio T., Tang J. (2015). Searching for Drug Synergy in Complex Dose-Response Landscapes Using an Interaction Potency Model. Comput. Struct. Biotechnol. J..

[25] Zheng S., Wang W., Aldahdooh J., Malyutina A., Shadbahr T., Tanoli Z., Pessia A., Tang J. (2022). SynergyFinder Plus: Toward Better Interpretation and Annotation of Drug Combination Screening Datasets. Genom. Proteom. Bioinform..

[26] Golay J., Semenzato G., Rambaldi A., Foà R., Gaidano G., Gamba E., Pane F., Pinto A., Specchia G., Zaja F. (2013). Lessons for the clinic from rituximab pharmacokinetics and pharmacodynamics. MAbs.

[27] Habermann T.M., Weller E.A., Morrison V.A., Gascoyne R.D., Cassileth P.A., Cohn J.B., Dakhil S.R., Woda B., Fisher R.I., Peterson B.A. (2006). Rituximab-CHOP versus CHOP alone or with maintenance rituximab in older patients with diffuse large B-cell lymphoma. J. Clin. Oncol..

[28] de Jong M.R.W., Langendonk M., Reitsma B., Nijland M., Berg A.v.D., Ammatuna E., Visser L., van Meerten T. (2019). Heterogeneous Pattern of Dependence on Anti-Apoptotic BCL-2 Family Proteins upon CHOP Treatment in Diffuse Large B-Cell Lymphoma. Int. J. Mol. Sci..

[29] Arribas A.J., Napoli S., Cascione L., Sartori G., Barnabei L., Gaudio E., Tarantelli C., Mensah A.A., Spriano F., Zucchetto A. (2022). Resistance to PI3Kdelta inhibitors in marginal zone lymphoma can be reverted by targeting the IL-6/PDGFRA axis. Haematologica.

[30] Arribas A.J., Napoli S., Cascione L., Barnabei L., Sartori G., Cannas E., Gaudio E., Tarantelli C., Mensah A.A., Spriano F. (2024). ERBB4-Mediated Signaling Is a Mediator of Resistance to PI3K and BTK Inhibitors in B-cell Lymphoid Neoplasms. Mol. Cancer Ther..

[31] Arribas A.J., Guidetti F., Cannas E., Cascione L., Napoli S., Sartori G., Fuzio F., Pesenti E., Tarantelli C., Spriano F. (2025). IL-16 production is a mechanism of resistance to BTK inhibitors and R-CHOP in lymphomas. bioRxiv.

[32] Arribas A., Napoli S., Cascione L., Gaudio E., Bordone-Pittau R., Barreca M., Sartori G., Chiara T., Spriano F., Rinaldi A. (2020). Secondary resistance to the PI3K inhibitor copanlisib in marginal zone lymphoma. Eur. J. Cancer.

[33] Silkenstedt E., Salles G., Campo E., Dreyling M. (2024). B-cell non-Hodgkin lymphomas. Lancet.

[34] Sehn L.H., Salles G. (2021). Diffuse Large B-Cell Lymphoma. N. Engl. J. Med..

[35] Armitage J.O., Longo D.L. (2022). Mantle-Cell Lymphoma. N. Engl. J. Med..

[36] Tarantelli C., Wald D., Munz N., Spriano F., Bruscaggin A., Cannas E., Cascione L., Gaudio E., Arribas A.J., Manjappa S. (2024). Targeting CD19-positive lymphomas with the antibodydrug conjugate loncastuximab tesirine: Preclinical evidence of activity as a single agent and in combination therapy. Haematologica.

[37] Kawasaki N., Nishito Y., Yoshimura Y., Yoshiura S. (2022). The molecular rationale for the combination of polatuzumab vedotin plus rituximab in diffuse large B-cell lymphoma. Br. J. Haematol..

[38] Simone B.A., Champ C.E., Rosenberg A.L., Berger A.C., A Monti D., P Dicker A., Simone N.L. (2013). Selectively starving cancer cells through dietary manipulation: Methods and clinical implications. Future Oncol..

[39] O’Flanagan C.H., Smith L.A., McDonell S.B., Hursting S.D. (2017). When less may be more: Calorie restriction and response to cancer therapy. BMC Med..

[40] Antunes F., Erustes A.G., Costa A.J., Nascimento A.C., Bincoletto C., Ureshino R.P., Pereira G.J.S., Smaili S.S. (2018). Autophagy and intermittent fasting: The connection for cancer therapy?. Clinics.

[41] Qin J.Z., Xin H., Nickoloff B.J. (2010). 3-Bromopyruvate induces necrotic cell death in sensitive melanoma cell lines. Biochem. Biophys. Res. Commun..

[42] Calviño E., Estañ M.C., Sánchez-Martín C., Brea R., de Blas E., Boyano-Adánez M.d.C., Rial E., Aller P. (2014). Regulation of death induction and chemosensitizing action of 3-bromopyruvate in myeloid leukemia cells: Energy depletion, oxidative stress, and protein kinase activity modulation. J. Pharmacol. Exp. Ther..

[43] Valenti D., Vacca R.A., de Bari L. (2015). 3-Bromopyruvate induces rapid human prostate cancer cell death by affecting cell energy metabolism, GSH pool and the glyoxalase system. J. Bioenerg. Biomembr..

