# Supplementary material for: KAT/3BP: A Metabolism-Targeting Agent with Single and Combination Activity in Aggressive B-Cell Lymphomas
Source: Cancers (Basel). 2025 Jun 18;17(12):2034. doi: 10.3390/cancers17122034 (PMC12191035; doi:10.3390/cancers17122034)
Supplement: Supplementary file 1 [file cancers-17-02034-s001.zip › cancers-3647702-supplementary.pdf]

The anti-metabolite KAT/3BP has *in vitro* and *in vivo* anti-tumor activity in lymphoma models.

Chiara Tarantelli * [1], Filippo Spriano * [1], Elisa Civanelli [1], Luca Aresu [2], Giorgia Risi [1], Eleonora Cannas [1], Omar Kayali [1], Luciano Cascione [1,3], Alberto J. Arribas [1,3], Anastasios Stathis [4,5], Young H. Ko [6], Francesco Bertoni [1,4]

[1] *Institute of Oncology Research, Faculty of Biomedical Sciences, USI, Bellinzona, Switzerland;*

[2] *Department of Veterinary Sciences, University of Turin, Grugliasco, Turin, Italy;*

[3] *SIB Swiss Institute of Bioinformatics, Lausanne, Switzerland;*

[4] *Oncology Institute of Southern Switzerland, Ente Ospedaliero Cantonale, Bellinzona, Switzerland;*

[5] *Faculty of Biomedical Sciences, USI, Lugano, Switzerland;*

[6] *KoDiscovery, LLC, Columbus Center/IMET, Baltimore, Maryland, USA.*

*equally contributed

Supplementary figures and tables

Supplementary Figure S1. The activity of KAT/3BP on lymphoma cell lines, one diffuse large B cell lymphoma (TOLEDO), and one mantle cell lymphoma (Z138). (A) Cell cycle distribution by Propidium Iodide (PI) staining and (B) apoptosis induction by Annexin V/PI FACS acquisition were assessed after 24, 48, and 72 hours of 5 mM drug concentration. Average of two independent experiments.

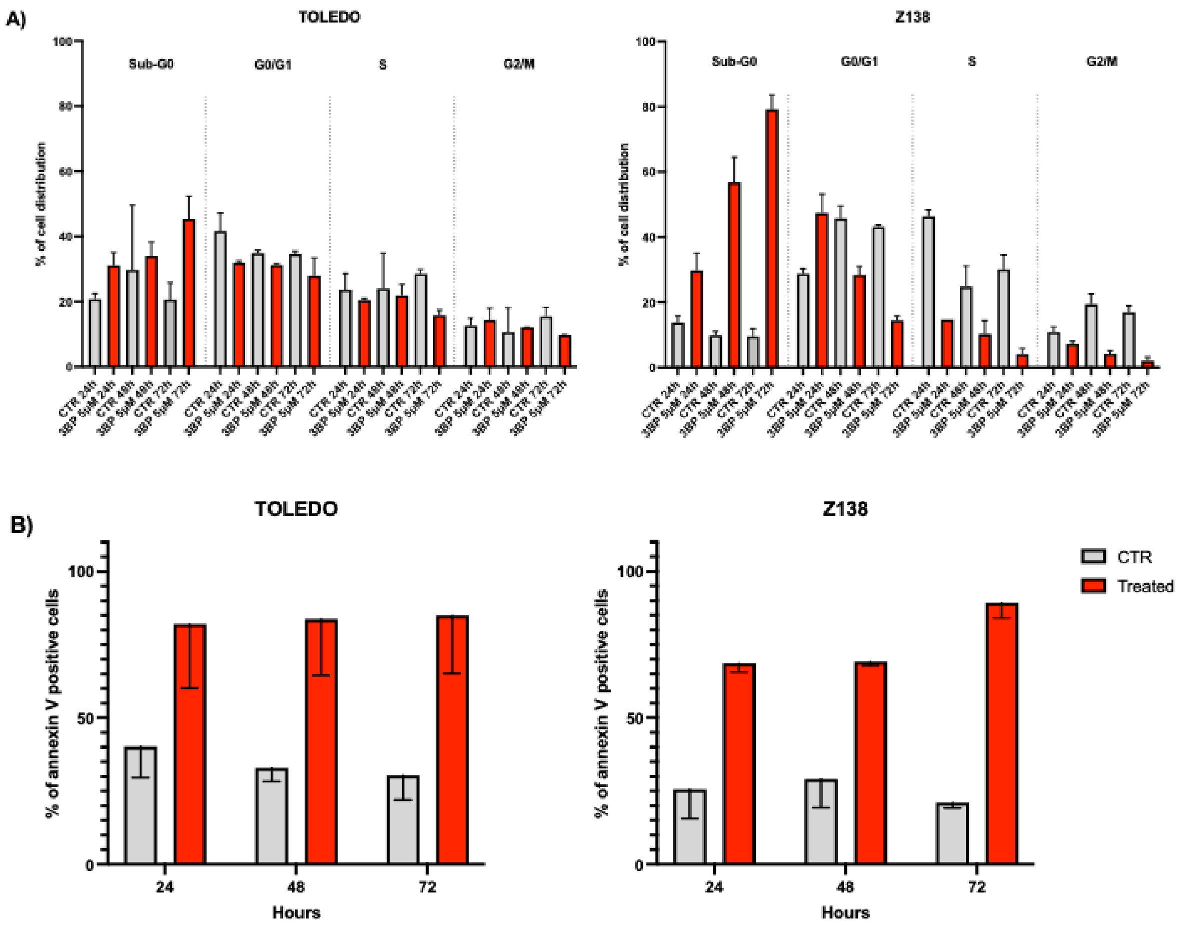

Supplementary Figure S2. Antiproliferative effect of 3BP in A20 murine cell lymphoma model. MTT assay was performed to evaluate the anti-tumoral activity of the drug after 72h of treatment.

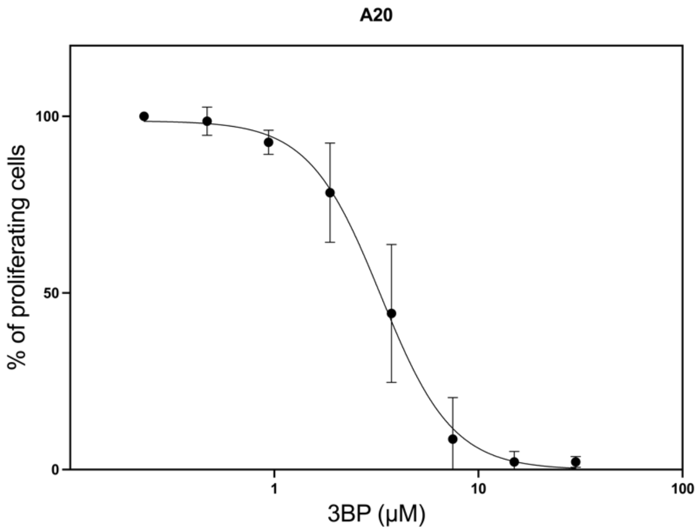

Supplementary Figure S3. Assessment of KAT/3BP anti-lymphoma activity in *in vivo* syngeneic model. A) Tumor volume mean with positive and negative SEM for each group. B) Body weight mean with standard deviation for each group. Figure up to the end of the experiment (day 92).

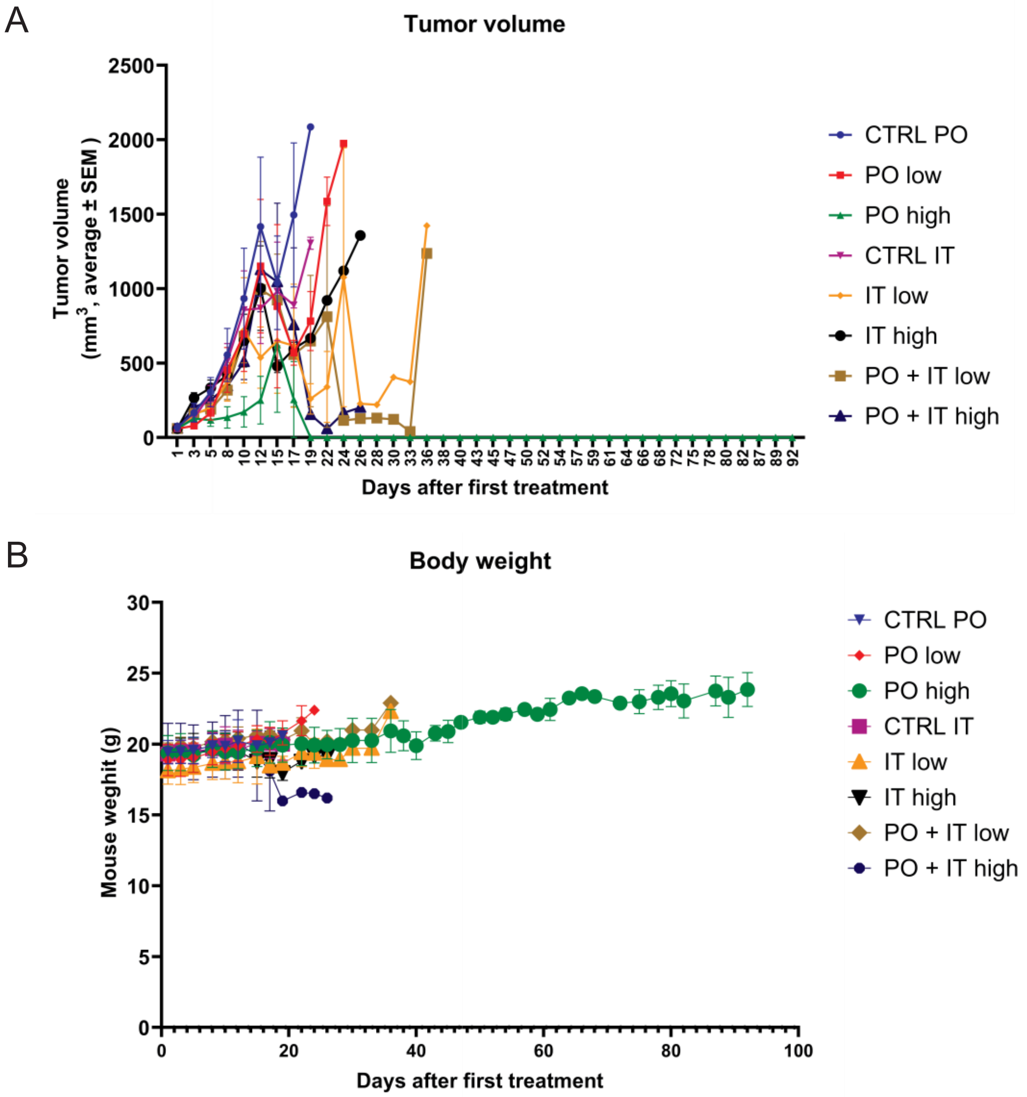

Supplementary Figure S4. Histological representation of tumor necrosis scores. A) Score 0: no necrosis present. The tumor tissue shows intact cellular architecture without evidence of cell death or tissue breakdown. B) Score 1: The focal area of cellular degradation and tissue disruption are visible, affecting up to 10% of the examined field. C) Score 2: more extensive areas of cell death and tissue disorganization are apparent. D) Score 3: widespread necrotic regions are evident, characterized by extensive cellular debris, loss of nuclear detail, and disrupted tissue architecture.

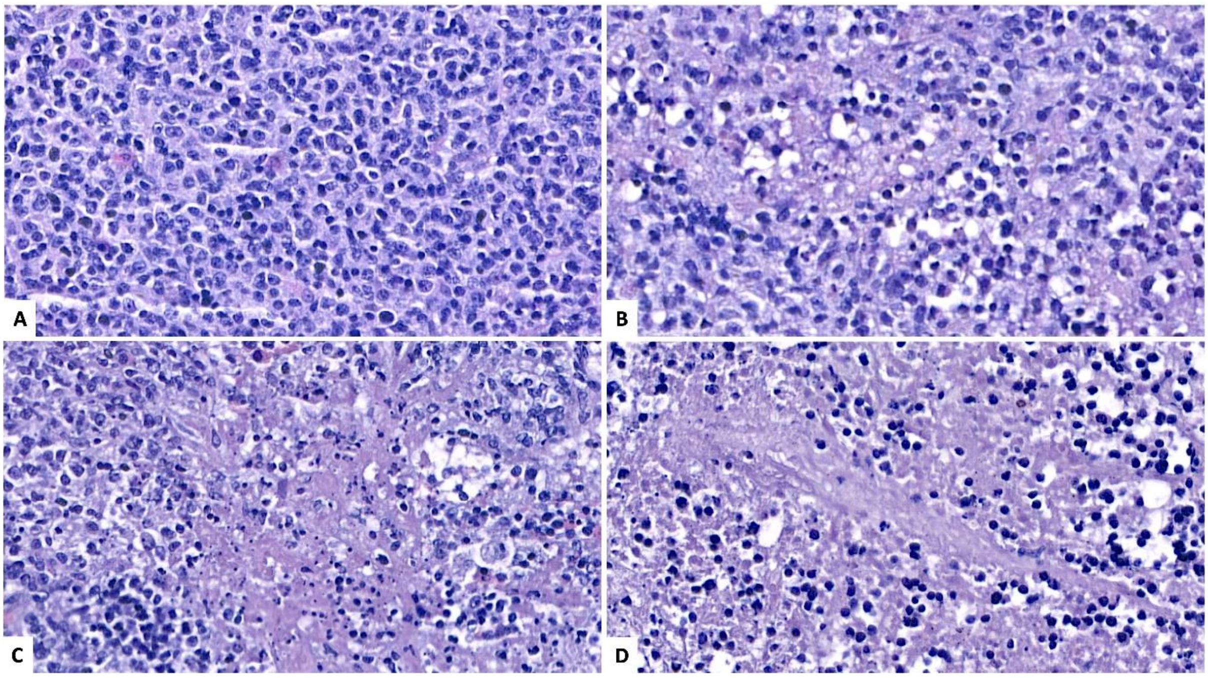

Supplementary Figure S5. Activity of KAT/3BP administered by intraperitoneal injection in a syngeneic model. BALB/c mice were subcutaneously injected with murine lymphoma cell line A20. Mice were treated with vehicle (SFB) by intraperitoneal (IP) injection, with 2.5 mg/kg and 10 mg/kg (low and high IP, respectively). A) Graphs showing tumor volume in mm^3 for each animal in each group. B) Probability of survival for each group. C) Body weight mean with standard deviation for each group. Figure up to the end of the experiment (day 26).

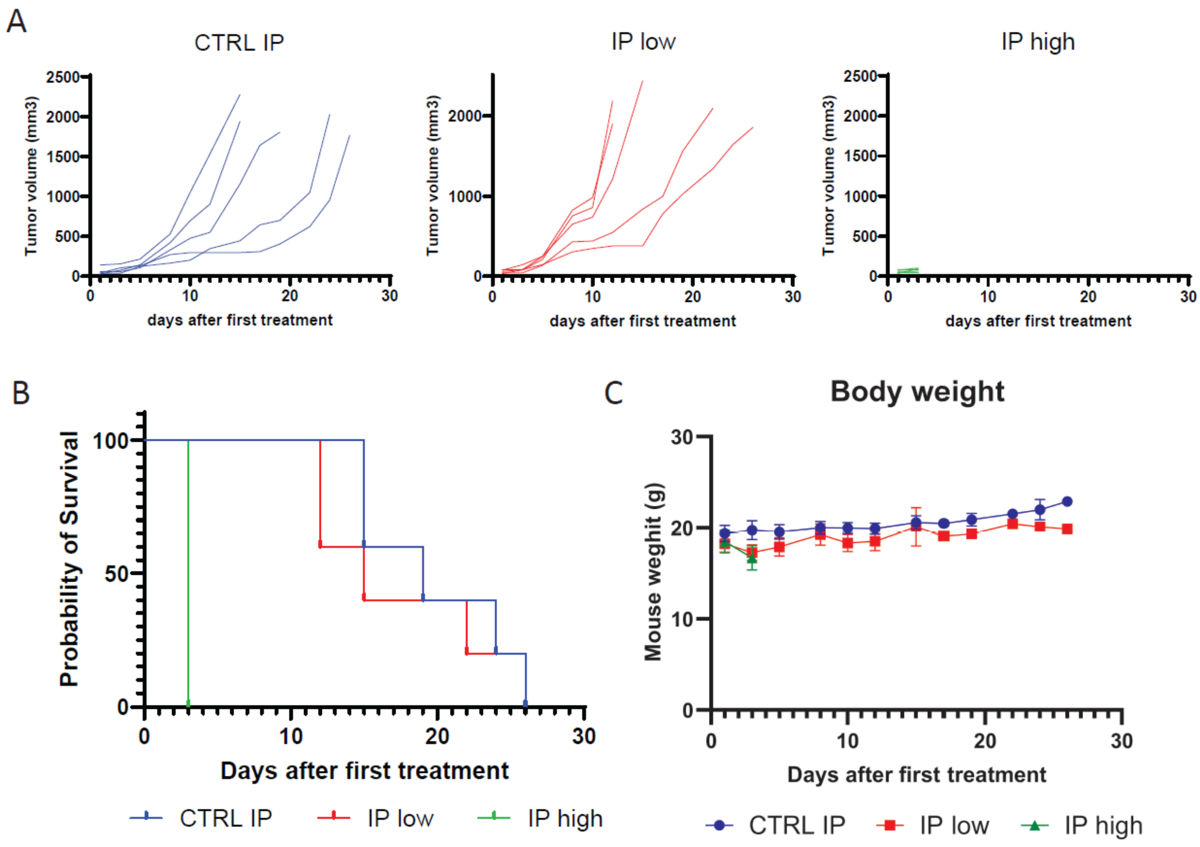

Supplementary Figure S6. Assessment of KAT/3BP anti-lymphoma activity in PO and IT administration as single and in combination. A) Tumor volume mean with positive and negative SEM for each group. B) Body weight mean with standard deviation for each group. Figures up to the end of the experiment (day 44).

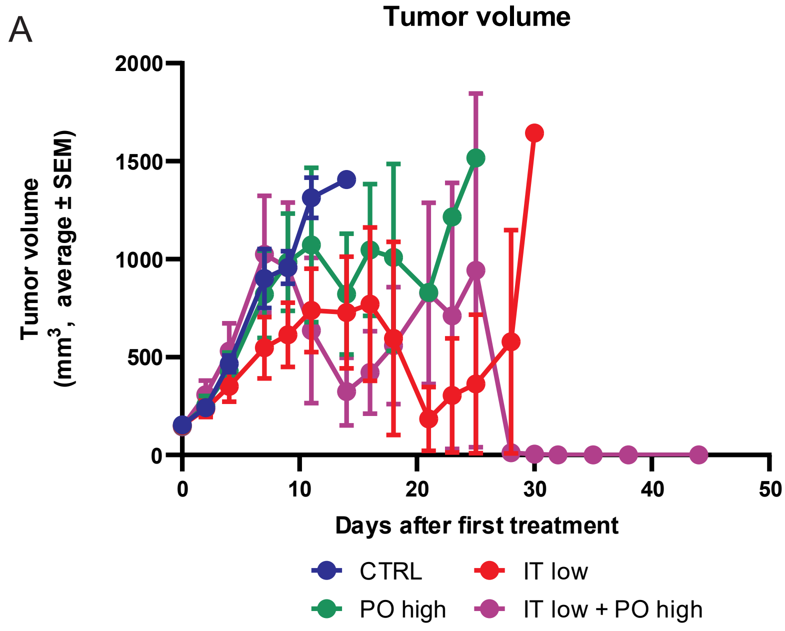

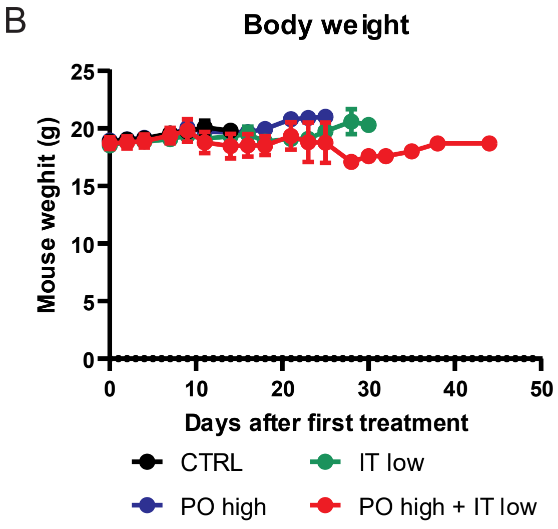

Supplementary Table S1. Compounds combined with KAT/3BP with their respective target and range of concentrations used. KAT/3BP was used at 30 µM 1:2 dilution. *, rituximab, cyclophosphamide, doxorubicin, vincristine, prednisone; ** in ABC-DLBCL, due to the drug clinical use; *** in GCB-DLBCL, due to the drug clinical use, and in the murine cell.

Agent	Mechanism of action	Concentration range
Bendamustine	Chemotherapy	20 µM (1:2 dilution)
R-CHOP *	Chemotherapy plus anti-CD20 monoclonal antibody	R, 20 µg/ml; CHOP, 2 µg/ml (1:2 dilution)
Venetoclax	BCL2 inhibitor	10 µM (1:4 dilution)
Ibrutinib **	BTK-inhibitor	20 nM TMD8; 20 µM 1:2 U2932 (1:2 dilution)
Lenalidomide **	Immunomodulatory	20 µM (1:2 dilution)
Copanlisib	pan class I PI3K inhibitor (PI3Kδ/α)	10 µM (1:4 dilution)
Tazemetostat ***	EZH2 inhibitor	25 µM (1:2 dilution
Vorinostat	HDAC inhibitor	10 µM (1:3 dilution)

Supplementary Table S2. Overall experimental design to assess the efficacy of KAT/3BP via various delivery routes (oral, IT, and IP) in tumor-bearing syngeneic mice.

Group n.	Total number of mice	compound	treatment	schedule	route
1	5	Vehicle (SFB)	/	4 on 3 off, 4 weeks, 1 W recovery	PO
2	5	Vehicle (SFB)	/	4 on 3 off, 4 weeks, 1 W recovery	IT
3	5	Vehicle (SFB)	/	4 on 3 off, 4 weeks, 1 W recovery	IP
4	5	KAT/3BP	2.5 mg/kg	4 on 3 off, 4 weeks, 1 W recovery	PO (low)
5	5	KAT/3BP	10 mg/kg	4 on 3 off, 4 weeks, 1 W recovery	PO (high)
6	5	KAT/3BP	0.5 mM	4 on 3 off, 4 weeks, 1 W recovery	IT (low)
7	5	KAT/3BP	2 mM	4 on 3 off, 4 weeks, 1 W recovery	IT (high)
8	5	KAT/3BP	2.5 mg/kg + 0.5 mM	4 on 3 off, 4 weeks, 1 W recovery	PO (low) + IT (low)
9	5	KAT/3BP	10 mg/kg + 2 mM	4 on 3 off, 4 weeks, 1 W recovery	PO (high) + IT (high)
10	5	KAT/3BP	2.5 mg/kg	4 on 3 off, 4 weeks, 1 W recovery	IP (low)
11	5	KAT/3BP	10 mg/kg	4 on 3 off, 4 weeks, 1 W recovery	IP (high)

Supplementary Table S3. Overall experimental design to assess the efficacy of KAT/3BP via various delivery routes in combination (PO, IT, combination) in tumor-bearing syngeneic mice.

Group n.	Total number of mice	compound	treatment	schedule	route
1	8	Vehicle (SFB)	/	4 on 3 off, 4 weeks, 1 W recovery	PO+IT
2	8	KAT/3BP	PO high 10.0 mg/kg	4 on 3 off, 4 weeks, 1 W recovery	PO high
3	8	KAT/3BP	IT low 0.5 mM	4 on 3 off, 4 weeks, 1 W recovery	IT low
4	8	KAT/3BP	PO high + IT low	4 on 3 off, 4 weeks, 1 W recovery	PO+IT
